# Spatial and Temporal Patterns of Eastern Australia Subtropical Coral Communities

**DOI:** 10.1371/journal.pone.0075873

**Published:** 2013-09-13

**Authors:** Steven J. Dalton, George Roff

**Affiliations:** 1 Marine Ecology Research Centre, Southern Cross University, Coffs Harbour, New South Wales, Australia; 2 School of Biological Sciences, University of Queensland, St Lucia, Queensland, Australia; The Australian National University, Australia

## Abstract

Despite increases in the frequency and intensity of disturbances on coral reefs over the past few decades, the response of subtropical coral assemblages to climate change is poorly understood. To address this knowledge gap on Australian reefs and provide a baseline for future comparisons, we quantified spatial (10-100’s of kilometres) and temporal (decadal) patterns of benthic assemblages across a latitudinal gradient along the east Australian coastline (23.5° S to 31.5° S). Benthic community composition was quantified at six locations from the southern Great Barrier Reef, Queensland (Heron Reef, 23.5° S, 152° E) to northern New South Wales (31° S, 153.1° E) and at Lord Howe Island (31.5° S, 159.1° E). Our results indicate significant latitudinal differences in benthic assemblages, while community composition at some sites was more similar to those hundreds of kilometres away than to that of neighbouring reefs. A general trend was observed with decreasing cover of Acroporidae with increasing latitude, corresponding with an increasing cover of Pocilloporidae and Dendrophylliidae. Heron Reef comprised a high proportion of *Acropora* corals (43% total coral cover) and coralline algae (44%). In contrast, high-latitude reefs were dominated by mixed coral assemblages (0-52%) and high macroalgal cover (16-27%). Decadal comparisons of high-latitude reefs showed regional stability of benthic assemblages (9 out of 11 assemblages remained stable at > 75% similarity), during a period of warming oceans (0.15-0.24°C per decade). Such temporal stability suggests that eastern Australian subtropical communities may be more resistant than tropical reef communities that have experienced assembly shifts caused by perturbations associated with recent global climate change. Despite the clear differences in the structure of coral assemblages evident in our spatial surveys, we suggest that the temporal stability of high-latitude reefs may provide a limited refuge for tropical coral populations in an increasingly uncertain future.

## Introduction

Scleractinian coral communities are exposed to a range of disturbances from both natural and human induced processes that can range from small-scale localised mortality events (e.g. predation and competition) to region-wide mortality events (e.g. disease outbreaks and coral bleaching) [1,2,3,4]. Recent large-scale loss in hard coral cover has led to a plethora of studies on the resilience of hard corals to a range of recent climate change associated perturbations at tropical locations [5,6,7]; however, there is a dearth of studies that detail the ability of corals to persist on marginal, high-latitude subtropical reefs under future climate change trajectories.

Coral assemblages along the eastern Australian continent extend from 11° S in the northern Great Barrier Reef (GBR) to as far south as 31° S along the coastline of New South Wales (NSW). On subtropical reefs adjacent to the NSW mainland, corals generally do not form accreting reef structures; rather colonies form a thin veneer attached to rocky substratum [8]. An exception are the high-latitude subtropical coral reefs found along the Lord Howe Rise, located 600 km east of mainland Australia, where active reef accretion is observed on the western side of Lord Howe Island [9,10]. In general, coral communities south of the GBR comprise a suite of tropical, subtropical and temperate species, with species richness declining with increasing latitude [11]. While low in diversity, coral cover at some sites is comparable to that of the GBR [12], with up to 45% cover observed at some locations [8,10,13,14,15].

In the past 30 years, the east coast of Australia has experienced significant warming trends of between 0.1-0.5°C per decade [16], raising concern about the future of tropical coral assemblages on the GBR [12]. Under future climate change scenarios, it has been suggested that high-latitude marginal reefs may provide refuge for tropical coral species [6,17,18]. This has been the case in historic times of climate change; in the Pleistocene, equatorial declines in reef corals were accompanied by range shifts and expansion towards subtropical latitudes [19,20]. However, subtropical eastern Australia marginal reefs are characterised by high macroalgal cover and low rates of coral recruitment [21], which may render these low resilient environments highly susceptible to future disturbances such as rising seawater temperature.

To date, several studies on eastern Australia high-latitude reefs have characterised coral communities over small spatial scales [8,10,13,14]. High-latitude reefs are typically characterised by an absence of reef building taxa (e.g. staghorn *Acropora* and massive *Porites*) that are commonly found at lower latitudes on the GBR, and higher levels of macroalgal cover [22]. However, a recent study has documented the increase in frequency of tropical species in high-latitude environments (e.g. *Acroporamicroclados* [18]). The observation of tropical species ‘appearing’ in marginal locations potentially indicates a change in species ranges driven by changes in ocean temperatures following climate change. Despite increases in disturbance events in subtropical reefs over the past few decades [23,24,25,26,27] and the potential for range shifts in coral taxa from lower latitudes, little is known about the temporal stability of high-latitude refugia, and few baselines exist with which to compare future shifts in coral community structure.

Through detailed surveys of subtropical reefs across a latitudinal gradient on the eastern Australian coastline (23.5° S to 31.5° S), we aimed to quantify coral assemblages across a range of spatial scales (local - regional scales). Additionally, a subset of our data is compared with earlier studies [13,14,15,28] to quantify temporal changes in subtropical environments. Specifically, we aimed to: 1) determine spatial patterns of benthic community structure among subtropical eastern Australia reefs; 2) contrast benthic assemblages across a latitudinal gradient exceeding 1000 km (southern GBR to northern NSW); and 3) quantify decadal trends in benthic community structure among eastern Australian subtropical reefs.

## Materials and Methods

### Study Sites

**Figure 1:**
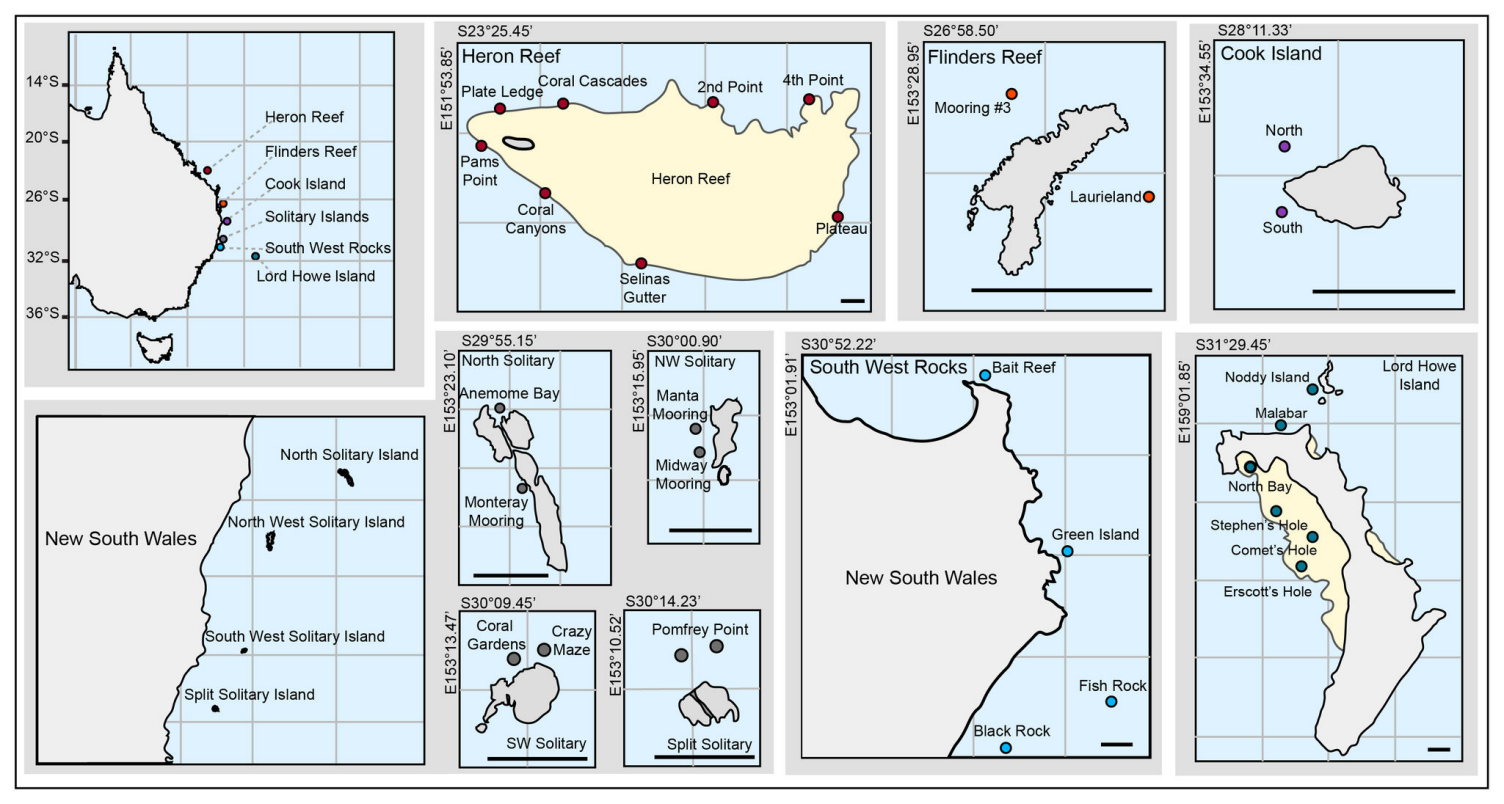
Map of study sites on the eastern Australian coastline. Site specific locations inset.

Subtropical benthic community composition data were collected from five locations along the east coast of Australia in 2006 (Figure 1): Flinders Reef (FR); Cook Island (CI); Solitary Islands Marine Park (SIMP); South West Rocks (SWR); and Lord Howe Island Marine Park (LHIMP). These surveys were conducted in conjunction with coral health assessment investigations and therefore sampling was done in areas of highest coral cover. Location details for SIMP, SWR, and LHIMP sites have been described previously [26]. Sites at FR were located on the northwest and northeast side of a small emergent sandstone outcrop of approximately 500 m long and 250 m wide. Two sites were investigated on the western side of CI. To allow comparisons between subtropical and tropical locations, benthic assemblage data was gathered from eight sites around Heron Reef (HERON) in the southern GBR [29]. Surveys were conducted at depths where coral cover was highest (8-14 m), with the exception of the four LHIMP lagoon sites (2-5 m depth) and HERON Reef slope (4-6 m). All surveys were conducted in accordance with the NSW Department of Primary Industry scientific research permit (*P06/0064*).

### Benthic community structure

Consistent with previous research on eastern Australian subtropical reefs [30], we used video transects and *in-situ* assessments of coral taxa to identify and quantify benthic community structure. Five 30-m transects were randomly positioned at each site and, using a Sony mini-dv camera in a waterproof housing, a SCUBA diver swam along each transect at a constant speed (0.1 m s^-1^), approximately 50 cm above the substratum. Two transects from each site were used to compile a comprehensive list of benthic categories across all sites. These categories complemented classifications previously used at these locations [13,14,15], thus enabling direct comparisons. From the video footage, coral cover and community structure were quantified for each transect using Coral ,Point Count (CPCe [31]). To enable comparison between data collected from Heron Reef and from surveys conducted in the 1990s at subtropical reefs, benthic categories were merged to the highest common taxonomic level possible, thus standardising variables over larger spatial and temporal scales.

### Spatial patterns of community structure among subtropical reefs

Benthic and coral community data from all sites were square-root transformed prior to analysis, which reduced the skewness in the data and improved homogeneity of variances [32]. A resemblance matrix of similarities was calculated using the Bray-Curtis coefficient and the resulting resemblance matrix visualised using Principal Coordinates Analysis (PCO). To test for differences in benthic assemblage over small (hundreds of metres to kilometres) and large (> 100 km) spatial scales, a Permutational Analysis of Variance (PERMANOVA [33]) was performed with location as a fixed factor and sites nested within location. Both *a priori* and *post-hoc* comparisons tested for overall and site differences, with significance values determined from 9999 and 999 unrestricted permutations, respectively. All univariate and multivariate analyse were preformed using PRIMER 6 / PERMANOVA+ software.

### Community structure comparisons between subtropical reefs and the southern GBR

To compare patterns of community structure between subtropical locations and southern GBR, coral community structure data from high-latitude sites (FR, CI, SIMP, SWR, LHIMP) were aggregated to Family taxonomic level (i.e., Acroporidae, Pocilloporidae, Faviidae, Dendrophylliidae) and compared with southern GBR data (HERON [29]). Abundance data were square-root transformed and PCO biplots visually displayed the resemblance data. To determine which taxonomic groups contributed most to patterns of community structure, eigenvectors of the variables (taxonomic groups) were overlaid onto the resultant PCO biplot.

### Decadal patterns of change in subtropical benthic assemblages

Subtropical benthic and coral assemblage data were compared with data collected during the 1990s [13,14,15,28] at each of the five subtropical locations (FR, CI, SIMP, SWR, LHIMP) using multivariate procedures. Data collected during this study were combined to common taxa groups, thus enabling direct comparison. PCO was used to visually display the underlying Bray-Curtis resemblance matrix (as detailed in the previous section), and trajectories were overlaid to show relative changes in assemblage composition between decades within each site.

## Results

### Spatial patterns of benthic and coral community structure

**Figure 2:**
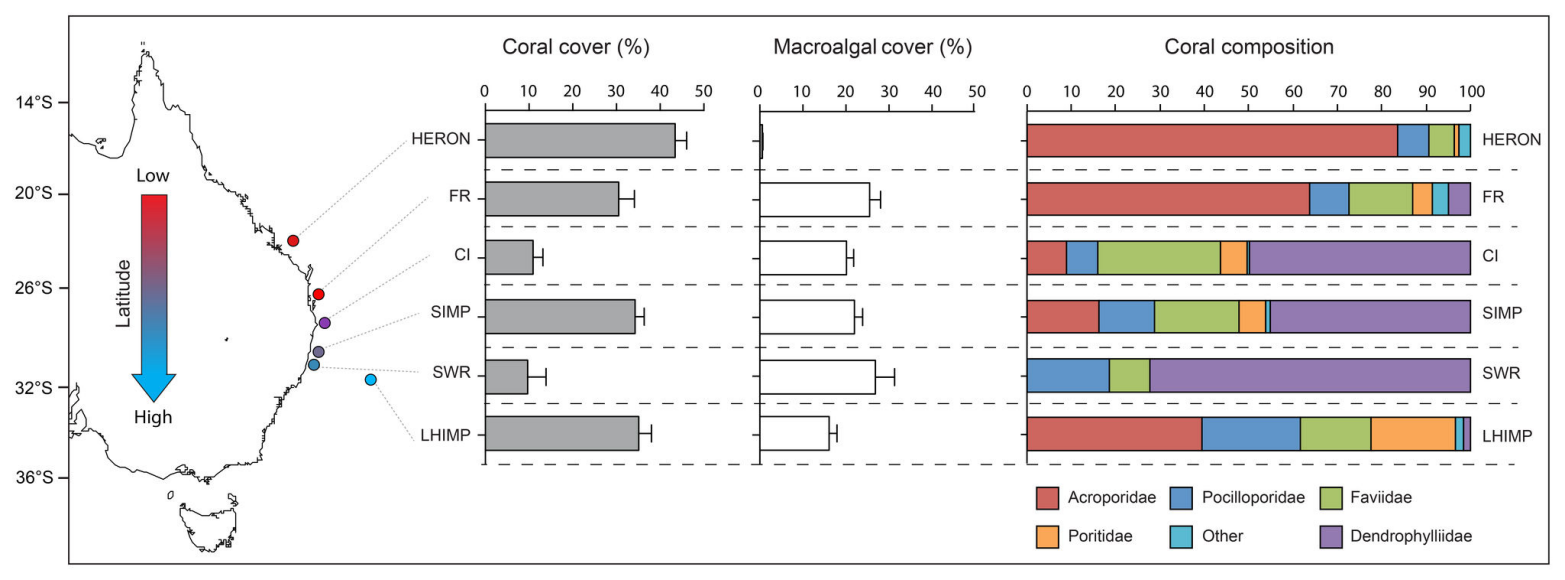
Latitudinal gradient of sites on the eastern Australian coastline. Corresponding latitudinal trends of coral cover, macroalgae and coral composition from sites.

Surveys of subtropical eastern Australian reefs and southern GBR showed that scleractinian corals were present at all locations, with the exception of two sites surveyed at SWR. Mean hard coral cover ranged between 0% at SWR sites and 52.3 ± 5.2% (± SE) at LHIMP (Figure 2). Macroalgal cover was relatively high across all subtropical reefs (16.1 ± 1.9% - 26.8 ± 4.5%), yet almost absent from surveys at the southern GBR sites (0.6 ± 0.1%, HERON). Clear latitudinal trends were observed with *Acropora* spp. declining with increasing latitude on reefs adjacent to the mainland (Heron to SWR) but not at LHIMP, where *Acropora* was the dominant coral genus. *Pocillopora* displayed a clear trend of increasing relative abundance with increasing latitude (Figure 2). In contrast, dendrophyliid species dominated nearshore (< 1 km) and midshelf (< 5 km) reefs.

**Figure 3:**
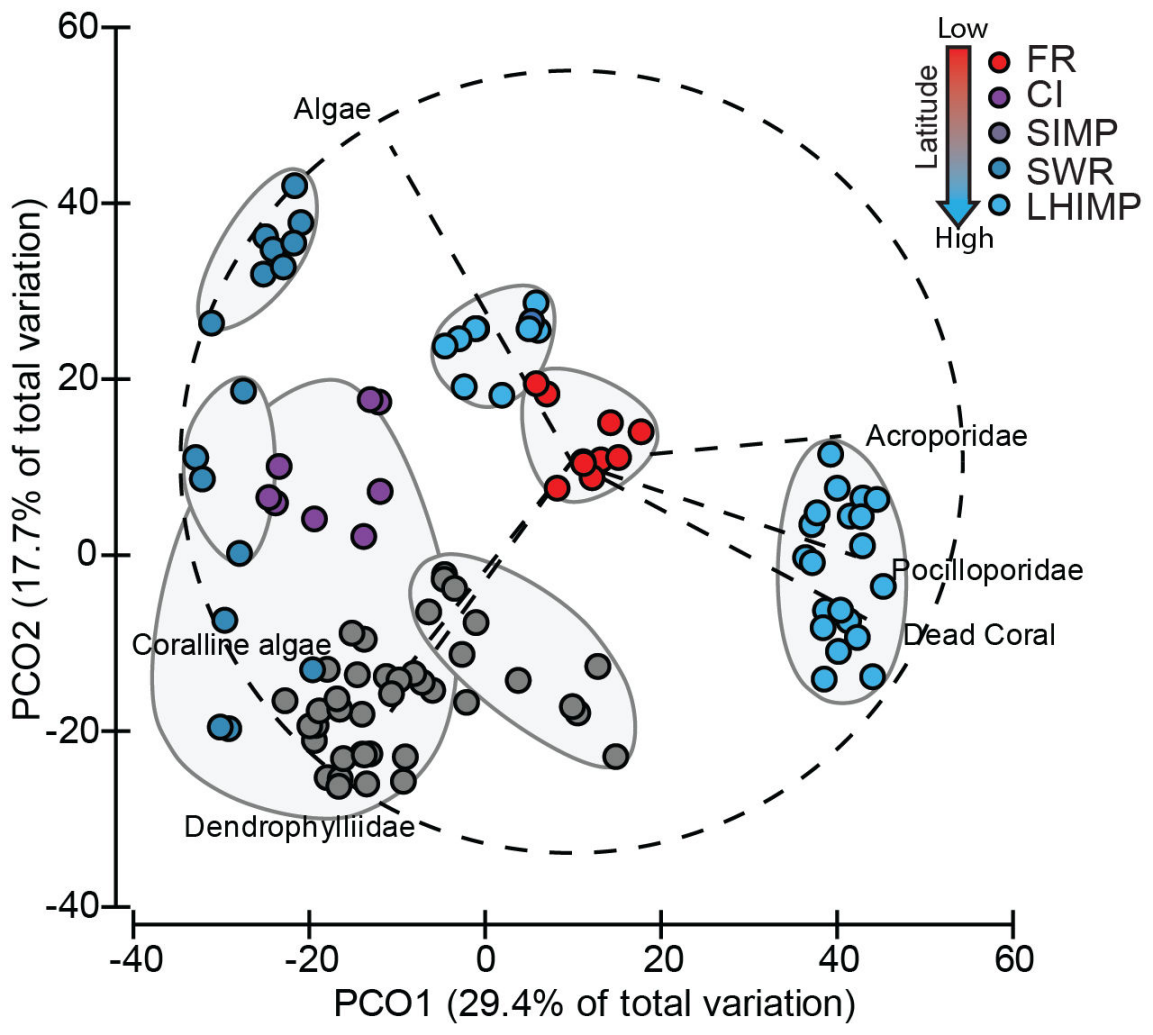
Principal coordinates analysis of benthic community composition from eastern Australia subtropical locations. Sites grouped by >65% similarity and Pearson correlation vectors overlaid to display benthic categories that contributed to greater than 70% correlation.

The principal coordinates analysis displayed a clear pattern of benthic community structure among subtropical locations, with the first and second axes explaining 29.4% and 17.7% of the total variation, respectively (Figure 3). Hierarchical cluster analysis of benthic taxa revealed strong clustering in six distinct groups, separated at the 65% similarity level. The coral Families Acroporidae and Pocilloporidae dominated LHIMP lagoon sites. Flinders Reef comprised a suite of coral species, including *Isopora* spp. *,* *Pocilloporadamicornis* and *Poritesheronensis* , thus orientating these sites toward the centre of the ordination. Bait Reef and Green Island (SWR) appeared distinct in the PCO; this dissimilarity was driven by the presence of high macroalgae cover (Figure 3). In contrast, Black Rock (SWR), most sites within the SIMP and CI grouped towards the bottom of the ordination and were distinguished from the other groups by the dominance of dendrophylliid species. Two-way PERMANOVA indicated that there was a highly significant difference in benthic assemblage between location (F_4, 103_ = 4.39, P = 0.001, ECV = 38.42%) and between sites within location (F_17, 103_ = 12.16, P = 0.001, ECV = 43.15%), suggesting that significant variability in community types exists both between and among high-latitude locations.

**Figure 4:**
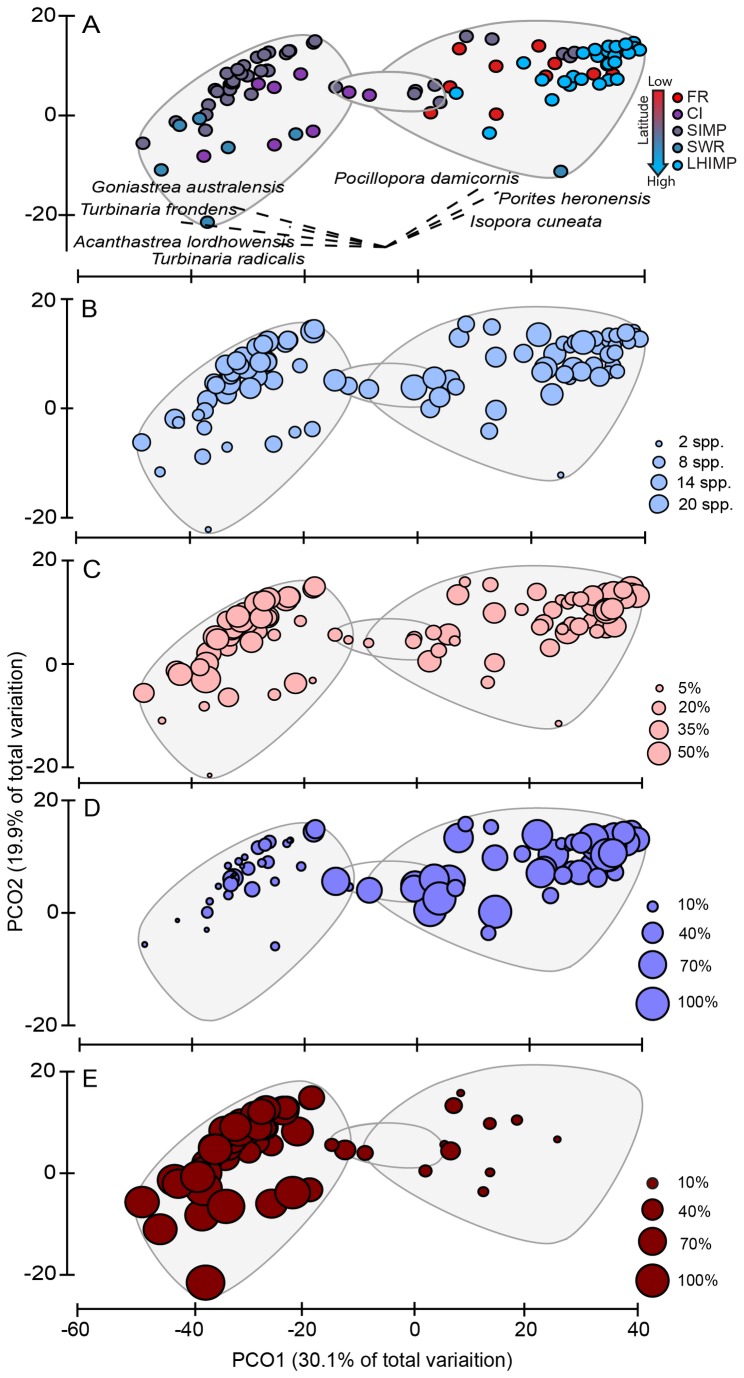
Principal coordinates analysis (PCO) of subtropical coral community composition along eastern Australia (A). Bubble plots overlaid onto PCO displaying B) mean coral taxa richness, C) mean percent coral cover, and D) proportion of Acroporidae and E) Dendrophylliidae cover of the total coral cover.

Clear patterns of coral community structure among locations were evident from the PCO (Figure 4A). Similarity cluster analysis of coral taxa showed a strong clustering in four distinct groups. Sites at LHIMP and FR were dominated by *Pocilloporadamicornis* , *Isopora* *spp.* and *Poritesheronensis* , while CI and SWR were dominated by *Turbinaria* spp., *Goniastreaaustralensis* and *Acanthastrea* species. A third cluster containing transects from CI and SIMP comprised an intermediate mix of coral taxa. Coral community structure at SIMP sites was highly variable, occurring in all three groups. No clear pattern was present within groups for coral generic richness (Figure 4B) nor coral cover (Figure 4C), in that groups with high and low diversity were present across all clusters, despite strong differences in the composition of taxa forming these clusters (Figure 4D, E). Acroporidae dominated the total coral cover at FR and LHIMP lagoon sites (right cluster, Figure 4D), while Dendrophylliidae comprised a high proportion of the coral assemblage in the sites that grouped to the left of the PCO (Figure 4E). Overall there were significant location (F_4, 103_ = 4.956, P = 0.001, ECV = 40.06%) and highly significant sites within location (F_17, 103_ = 8.78, P = 0.001, ECV = 37.18%) differences amongst the coral assemblage found on these reefs. *Post-hoc* analysis revealed significant variability in coral composition between SIMP and all other locations except CI; and between LHIMP and CI and SWR (Table 1).

**Table 1:** *Post-hoc* results from PERMANOVA, which compared coral community structure between subtropical locations.

	FR	CI	SIMP	SWR	LHIMP
FR	X	-	-	-	-
CI	ns	X	-	-	-
SIMP	*	ns	X	-	-
SWR	ns	ns	**	X	-
LHIMP	ns	*	***	***	X

X = no test, ns = not significant, ^*^ *p* < 0.05, ^**^ *p* < 0.01, ^***^ *p* < 0.001_._

**Figure 5:**
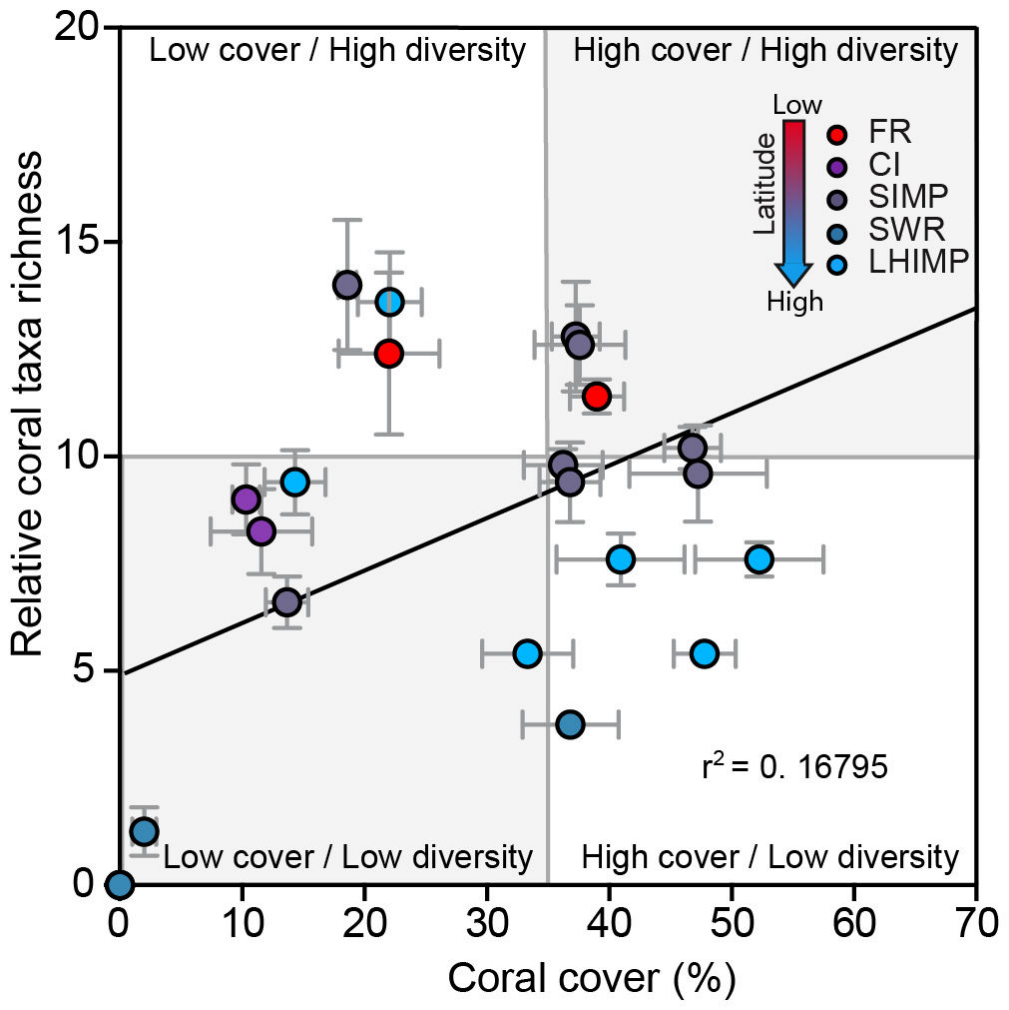
The relationship between relative coral taxa richness and coral cover recorded from replicate video transects recorded from subtropical reefs. Line represents a goodness-of-fit relationship based on linear regression model.

A weak but significant trend across all sites indicated that coral generic richness increased with percent coral cover (R^2^ = 0.168, F_1, 20_ = 4.037, *p* = 0.048; Figure 5). Sites at FR were characterised by high coral cover and mix of low/high diversity. SIMP sites were highly variable in coral cover and diversity. LHIMP lagoon sites tended to be high in coral cover with low diversity, while the LHIMP two deeper sites were characterised by low cover/high diversity and low cover/low diversity (Figure 5). CI and SWR sites were generally low in coral cover and diversity.

### Community structure comparisons between subtropical reefs and the southern GBR

**Figure 6:**
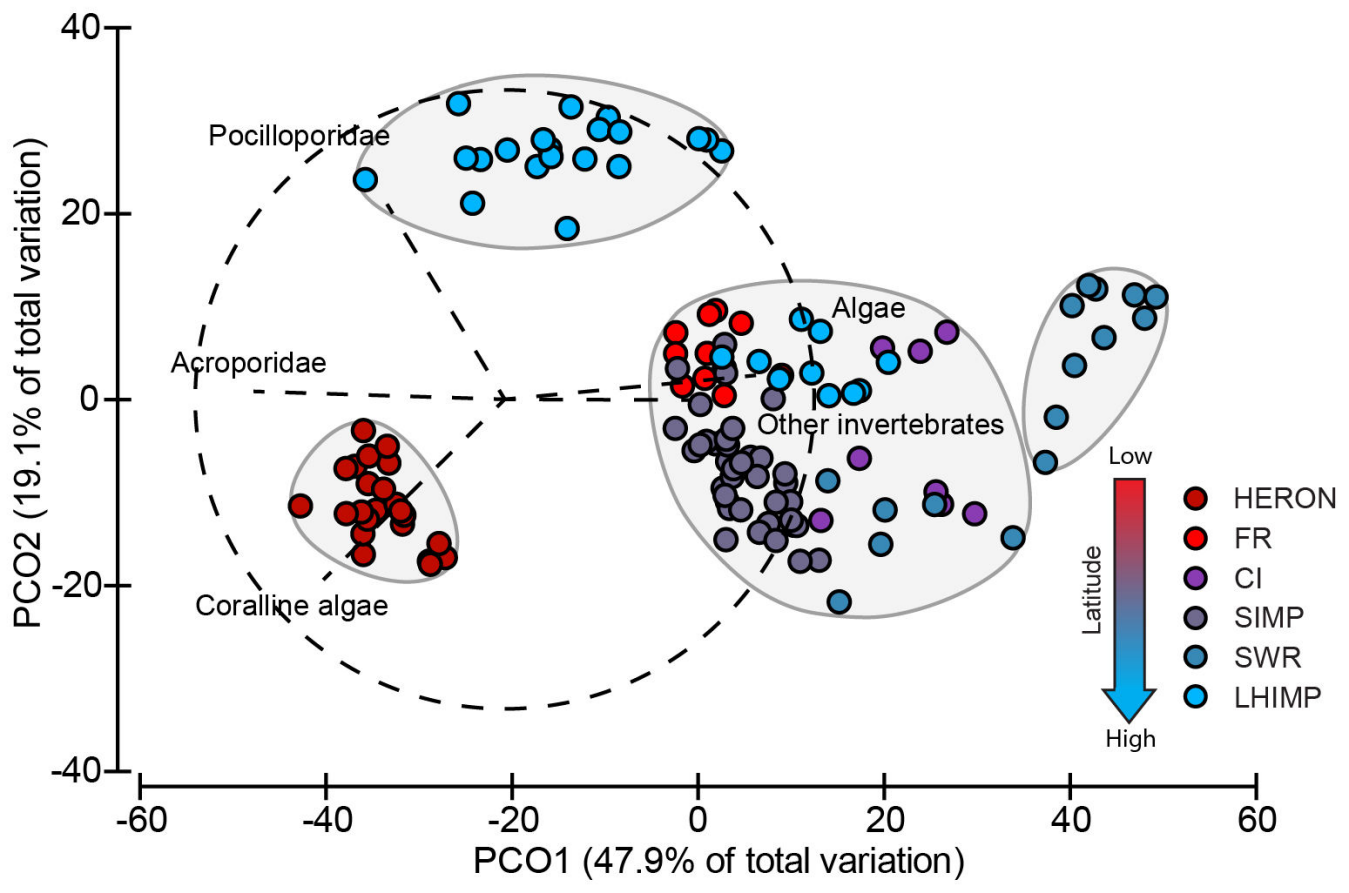
Principal coordinates analysis of the benthic community resemblance matrix compiled from reefs extending from southern GBR (Heron Reef) to subtropical (FR, CI, SIMP, SWR and LHIMP) locations. Main taxa vectors (dotted lines) that contributed greater than 75% correlation displayed and sites replicates grouped according to 75% similarity.

The first axis of the PCO for the benthic community resemblance matrix (Figure 6) explained 47.9% of the total variation, while the second axis explained 19.1%. Hierarchical cluster analysis revealed four distinct groups at the 65% resemblance level (Figure 6), separating HERON, LHIMP lagoon sites and SWR. HERON groups clustered towards vectors of coralline algae and Acroporidae, whereas LHIMP lagoon sites orientation demonstrated a dominance of Pocilloporidae. In contrast, SWR clustered strongly to the opposite side, dominated by other invertebrates and algae. The remaining subtropical sites formed the fourth group representing a similar centroid mix of community types.

PERMANOVA analysis indicated a significant effect for location and sites nested within location, which together explained the largest component of variance (28.76% and 26.89% ECV, respectively). Distance from mainland (18.35%) and latitude (17.71%) were also significant, but contributed to a lesser extent (Table 2). *Post-hoc* analysis of location indicates that HERON was significantly different from all subtropical sites (Table 3). SIMP was also significantly different from all other sites (Table 3). FR, CI and SWR were not significantly different from each other. LHIMP was significantly different from all sites except FR (Table 3).

**Table 2:** PERMANOVA results of benthic community structure.

**Source**	**df**	**SS**	**MS**	**Pseudo-F**	**P(perm**)	**perms**	**ECV**
Latitude	1	31104	31104	22.55	0.001	999	17.71
Distance	1	27264	27264	15.017	0.001	999	18.35
Location	5	25344	5068.8	3.2763	0.002	999	28.76
Sites (Location)	23	35904	1561.1	14.254	0.001	999	26.89
Res	96	10514	109.52				8.29
Total	126	1.30E+05					

Latitude and distance from mainland as covariates, site as a random factor nested in location, location as a fixed factor.

**Table 3:** *Post-hoc* results from PERMANOVA of benthic community structure between locations.

	HR	FR	CI	SIMP	SWR	LHIMP
HR	X	-	-	-	-	-
FR	*	X	-	-	-	-
CI	***	ns	X	-	-	-
SIMP	***	*	*	X	-	-
SWR	***	ns	ns	***	X	-
LHIMP	***	ns	*	***	***	X

X = no test, ns = not significant, ^*^ *p* < 0.05, ^**^ *p* < 0.01, ^***^ *p* < 0.001_._

### Decadal patterns in subtropical community structure

**Figure 7:**
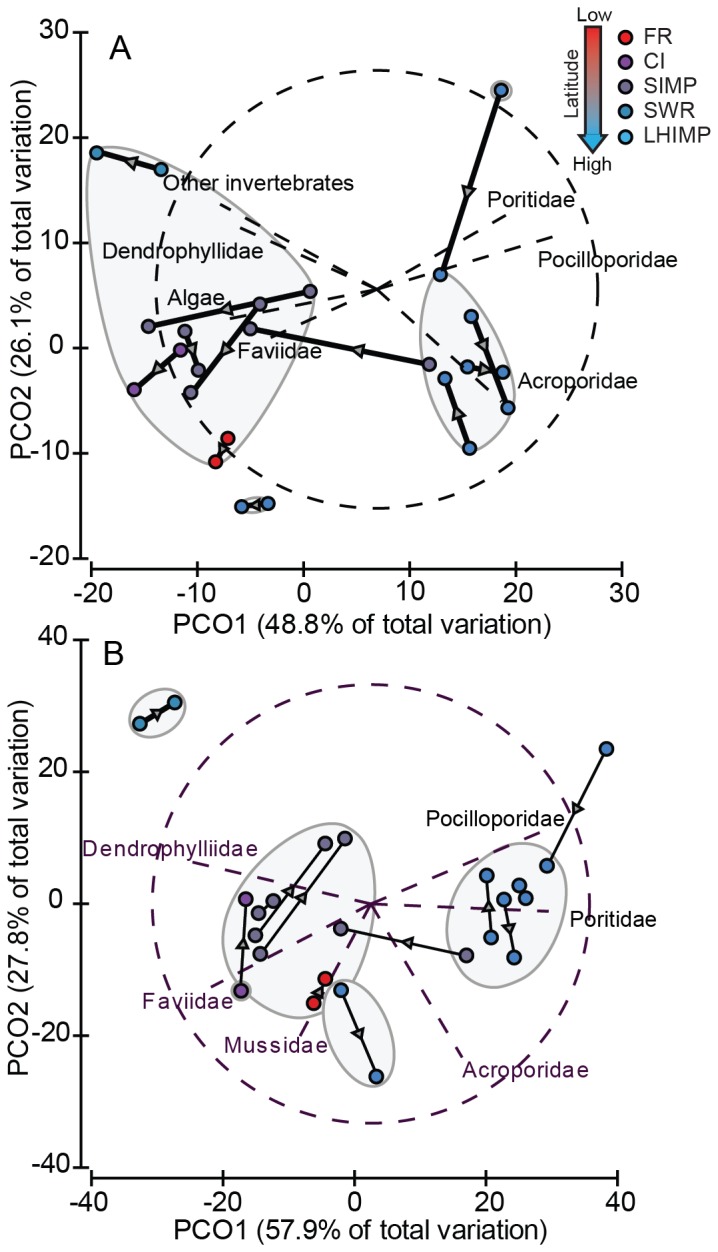
Decadal patterns of change in A) benthic and B) coral assemblages at five subtropical locations between the 1990s and 2000s. Sites compared included: FR – data pooled from two sites, Mooring 3 and Laurieland; CI – data pooled from two sites, North and South; SIMP – North, North West, South West and Split Solitary islands; SWR – Black Rock; and LHIMP – North Bay, Stephen’s Hole, Comet’s Hole, Erscott’s Hole and Malabar Reef.

The principle coordinates analysis of decadal changes in benthic and coral community composition resemblance matrices revealed that the first two eigenvectors accounted for 75% and 86% of the total similarities, respectively (Figure 7A, B). Cluster analysis indicated four distinct groups at the 75% similarity level for both data sets, indicating few differences among sites. PERMANOVA also revealed no significant decadal effect on both benthic and coral pattern of assemblage, during a period when seawater temperatures have risen between 0.15 and 0.24°C per decade over the past 30 years (Table S1). As shown by the site trajectories, only two sites deviated from the 75% similarity level (Figure 7A, B). One LHIMP site moved from a Poritidae/Pocilloporidae dominance to an Acroporidae dominance, while one SIMP site moved from Acroporidae dominance to macroalgae/Faviidae dominance. The remaining sites remained stable through time.

## Discussion

### Latitudinal variation in benthic community composition

Our results revealed significant regional differences in benthic community composition across a large latitudinal scale ranging from tropical to subtropical reef communities. Significant difference in community composition was found over small spatial scales (< 1 km), but our results also indicate remarkable similarities between locations that were separated by hundreds of kilometres. For example, the benthic community at FR (high cover of *Acropora* spp.) was more similar to some LHIMP sites than the geographically closer CI assemblage. In contrast, the benthic assemblage at CI was dominated by marine algae and was similar to LHIMP deeper and SIMP sites. This resemblance may be due to a number of physical factors including wave exposure and nutrient availability at these locations.

Benthic communities at CI, which is located approximately 4 km south east of a large coastal river (Tweed River), are regularly exposed to terrigenous discharge following storm events. The direction of the freshwater plume into the marine environment is determined by the strength of local wind, wave action and current direction, particularly the East Australian Current (EAC), which generally deflects riverine waters from the Tweed River to the south [34,35]. Additionally, the combination of the southward flowing EAC and the narrowing of the NSW continental shelf results in the formation of thermal fronts along the nearshore zone adjacent to Tweed Heads, Cape Byron and South West Rocks [36,37]. These fronts regularly transport cool upwelled, nutrient-rich waters in a northward direction, resulting in highly productive regions, and facilitate phytoplankton and macroalgal growth. Similarly, exposed reef communities at LHIMP are regularly subjected to dominant south to south-easterly sea conditions. This generates a dynamic environment with a constant supply of nutrients from northward flowing waters and upwellings associated with the Lord Howe Seamount [38].

Harriott and Banks [11] suggested that increased nutrients from periodic pulses of terrigenous discharge and upwelling events, stimulate algal growth along the NSW coast. Collectively, these factors may limit coral settlement while enhancing the growth of macroalgae and other competing sessile invertebrates. These competing organisms potentially affect coral calcification and growth (through bioerosion), limit coral recruitment (competition for space) and reduce the survival of new coral recruits, producing sub-optimal conditions for coral and reef growth in high-latitude reef environments on the eastern Australian coastline.

In high-latitude environments, exposure and wave energy play a significant role in structuring coral assemblages [39,40]. Along the northern NSW coast, wave height of the dominant south easterly seas can often exceed 6 m during unstable climatic conditions, with a maximum height of > 13 m being recorded in the past 10 years [41]. Previous studies have demonstrated a strong correlation between wave energy and coral distribution [42,43]. Jokiel et al. [44] demonstrated that coral cover, diversity and species richness in Hawaii were all negatively correlated with maximum wave height and wave direction. Considering the potential effects of such extreme wave exposure, further studies of small-scale wave exposure gradients would enable a clearer understanding of disturbance regimes and the distribution of benthic communities on subtropical reefs in eastern Australia.

### Subtropical reef coral community composition

Our results indicated that between location variability in coral community composition (40.06%) was greater than the variation within locations (37.18%). Such local-scale variability indicates considerable heterogeneity in high-latitude coral assemblages. For example, significant differences in community structure were observed among sites at SWR, despite the spatial separation of less than 4 km. The benthic community at Black Rock (SWR) was characterised by relatively high coral cover and diversity, which is more similar to sites at SIMP (70-100 km away) than it is to nearby sites within the same location (SWR). This may reflect local-scale variability in recruitment patterns, or the effects of small-scale variability in exposure and wave energy in structuring coral assemblages in high-latitude reefs. Our results also indicated that coral species richness at SWR was significantly lower than at all other locations. This abrupt decline in coral cover in combination with a significant reduction in species richness south of the SIMP may indicate the limited or sporadic connection between SWR and northern locations or represents the current biogeographical environmental limits of many subtropical and tropical coral species [11].

The higher abundance of *Acropora* spp. at NSI compared to other reefs in the SIMP may be due to the increased influence of the southward flowing EAC, which has been hypothesised to carry broadcast spawning *Acropora* coral larvae from northern reefs such as FR [45]. Seawater temperatures at NSI tends to be 1-2°C above those recorded at the midshelf islands of SIMP [46], which suggests a greater influence of the EAC and potentially provides a more suitable recruitment temperature for *Acropora* species arriving from northerly reefs. Wilson and Harrison [47] observed asynchronous spawning of 24 coral species at the northern islands in the SIMP and suggested the potential for successful recruitment of planulae larvae during favourable oceanic conditions. However, they indicated that recruitment at high-latitude reefs is restricted by the limited release of gametes, reduced fertilisation rates, and the sporadic nature of the dominant currents, which may restrict settlement of NSI spawned corals further south.

### Community structure comparisons between subtropical reefs and the southern GBR

Our results indicate clear differences in high-latitude subtropical community structure compared to the southern GBR site (Heron Reef). Multivariate correlation analysis showed a significant difference between southern GBR and high-latitude sites, with latitude, distance from shore, location and sites within location all having significant influence. That site nested within location was significant, even with latitude as a covariate within our model, suggests that other site-specific factors independent from latitude may be controlling the pattern of benthic community structure across sites. Our results indicate that location contributed greatest to the percent estimated components of variation, suggesting a strong difference between locations and a break in assemblage composition between southern GBR and locations further south.

Heron Reef sites comprised a high coral cover (mainly branching and tabular *Acropora* spp.) and coralline algae, with a virtual absence of macroalgae, which is typical of Indo-Pacific coral reefs [48]. The high cover of coralline algal cover recorded at Heron Reef may promote coral recruitment at this location [49], while the high rates of herbivory controls the levels of macroalgae and turf algae cover [50]. In contrast, high-latitude locations further south were characterised by lower coral cover and high macroalgal cover, with low abundance of coralline algae. Such high levels of macroalgal cover are likely to have a significant negative effect on the recruitment of corals, as macroalgae strongly suppress coral recruitment rates (e.g. [51,52]). Indeed, studies of coral recruitment at high-latitude sites (e.g. SIMP) recorded up to 35 times lower rates of coral recruitment compared to sites on the GBR [53]. This difference was primarily attributed to reduced light levels, low herbivore abundance, high macroalgal cover, and greater competition for settlement space with other benthic organisms that are more abundant at temperate locations (e.g. sponges and ascidians). Such consistently high levels of macroalgal cover and low rates of coral recruitment at high-latitude sites will likely have significant negative implications for the potential of these reefs to function as coral refugia (particularly for tropical coral species) under future climate change scenarios.

### Decadal patterns in subtropical community structure

Comparisons between this study and research completed during the early 1990s revealed that benthic and coral assemblages have remained stable in recent times, during a period when high-latitude reefs have been exposed to disease epizootics, bleaching conditions and storm events [25,26,54,55], and increases in sea surface temperatures of 0.19-0.24 °C per decade (Table S1). Despite high thermal stress at the locations recorded in the present study during the study period [24,56], benthic assemblages remained remarkably stable. Environmental factors governing high-latitude locations such as upwelling, strong currents, cloud cover, and light attenuation with increasing depth, have been reported to reduce the impact of thermal and light stress during bleaching events. These extrinsic factors appear to be acting independently or synergistically to mitigate conditions that induce thermal stress in high-latitude coral communities. This hypothesis is supported by a study of two subtropical regions (Bahamas and South Africa [57]), where cooler upwelled water minimised the effects of thermal stress on coral communities during the 1998 global mass bleaching event. Additionally, the authors proposed that adjacent deeper reefs would also provide a local larval supply to replenish damaged shallow areas following bleaching events [57].

Local upwelling and deep reefs are common features of benthic habitats along the coast of northern NSW, particularly throughout the SIMP. Recent swath mapping of seabed habitats has found reefs extending from mainland Australia to the continental shelf, and hard corals are a dominant component of midshelf exposed reefs between depths of 10-25 metres at 30° S [58]. Further genetics work on the connectivity of this region is needed to determine whether these deeper reefs may provide a refugium by acting as a local larval source for coral recruitment to shallower reefs following acute disturbances. Despite such potential for refugia during times of stress, intrinsic factors, such as limited genetic diversity in subtropical coral populations [59,60,61], reduced local reproductive capacity [47,62,63] and sporadic long-distance recruitment [53] may reduce the ability of subtropical coral communities to successfully recover from future chronic or catastrophic disturbances that result in a significant loss of coral cover.

The relative stability of subtropical coral communities at the sites studied contrasts the current trend of coral decline at tropical locations; approximately 20% and 80% of hard coral cover loss has occurred on Indo-Pacific and the Caribbean reefs, respectively [4,64,65]. This consistency of coral cover over the past 10-15 years on subtropical eastern Australian reefs, despite increases in sea surface temperature and the frequency of extreme warm events (Table S1 [16]) may indicate that these subtropical communities are resilient to perturbations associated with global climate change (e.g. bleaching and disease outbreaks), or that environmental change associated with climate change has not yet led to a decline in coral cover within this region. This subtropical region represents an area where a history of naturally variable environmental conditions (e.g. constant temperature fluctuation) may have led to greater resistance to elevated temperatures over geological time, and further investigation into these marginal coral communities is warranted.

## Supporting Information

Table S1 **Average changes in coastal sea surface temperatures (SST) between 1982 and 2010 at study locations (data for Gladstone, Flinders Reef, Cook Island, Solitary Islands, South West Rocks from Lima & Wethey 2012).** Average SST change per decade for Lord Howe Island was calculated from NOAA OI 1⁄4 Degree Daily SST Analysis data following the methods of Lima & Wethey (2012).
(DOCX)
