# Supplementary material for: Spatial and Temporal Patterns of Eastern Australia Subtropical Coral Communities
Source: PLoS One. 2013 Sep 13;8(9):e75873. doi: 10.1371/journal.pone.0075873 (PMC3772894; doi:10.1371/journal.pone.0075873)
Supplement: Table S1 — Average changes in coastal sea surface temperatures (SST) between 1982 and 2010 at study locations (data for Gladstone, Flinders Reef, Cook Island, Solitary Islands, South West Rocks from Lima & Wethey 2012). Average SST change per decade for Lord Howe Island was calculated from NOAA OI 1⁄4 Degree Daily SST Analysis data following the methods of Lima & Wethey (2012). (DOCX) [file pone.0075873.s001.docx]

**Table S1** Average changes in coastal sea surface temperatures (SST) between 1982 and 2010 at study locations (data for Gladstone, Flinders Reef, Cook Island, Solitary Islands, South West Rocks from Lima & Wethey 2012). Average SST change per decade for Lord Howe Island was calculated from NOAA OI 1⁄4 Degree Daily SST Analysis data following the methods of Lima & Wethey (2012).

| Location | Average SST Change (°C per decade ± SE) | Average change in # of annual extreme hot days | Average change in # of annual extreme cold days |
| --- | --- | --- | --- |
| Heron Island (Gladstone) | 0.15 ± 0.01 ^*^ | 7.4 ± 5.17 ^ns^ | -4.57 ± 5.44 ^ns^ |
| Flinders Reef | 0.21 ± 0.01 ^*^ | 8.6 ± 5.18 ^ns^ | -6.41 ± 4.78 ^ns^ |
| Cook Island | 0.19 ± 0.01 ^*^ | 10.22 ± 4.71 ^*^ | -7.12 ± 3.72 ^ns^ |
| Solitary Islands | 0.24 ± 0.01 ^*^ | 13.1 ± 5.66 ^*^ | -11.65 ± 4.39 ^*^ |
| South West Rocks | 0.22 ± 0.01 ^*^ | 14.63 ± 5.67 ^*^ | -9.57 ± 3.37  ^*^ |
| Lord Howe Island | 0.19 ± 0.01 ^*^ |  |  |
